# Faster recovery and bowel movement after early oral feeding compared to late oral feeding after upper GI tumor resections: a meta-analysis

**DOI:** 10.3389/fsurg.2023.1092303

**Published:** 2023-05-25

**Authors:** Dóra Lili Sindler, Péter Mátrai, Lajos Szakó, Dávid Berki, Gergő Berke, Armand Csontos, Csenge Papp, Péter Hegyi, András Papp

**Affiliations:** ^1^ Department of Surgery, Clinical Center, Medical School, University of Pécs, Pécs, Hungary; ^2^ Institute for Translational Medicine, Medical School, University of Pécs, Pécs, Hungary; ^3^ János Szentágothai Research Centre, Medical School, University of Pécs, Pécs, Hungary; ^4^ Department of Emergency Medicine, Medical School, University of Pécs, Pécs, Hungary; ^5^ First Department of Surgery, Military Hospital Medical Centre, Hungarian Defense Forces, Budapest, Hungary; ^6^ First Department of Medicine, Medical School, University of Szeged, Szeged, Hungary; ^7^ Hungary Centre for Translational Medicine, Semmelweis University, Budapest, Hungary; ^8^ Division of Pancreatic Diseases, Heart and Vascular Center, Semmelweis University, Budapest, Hungary

**Keywords:** early oral feeding (EOF), upper GI surgery, meta-analysis, upper GI cancer, Eras

## Abstract

**Background:**

There were more than 1 million new cases of stomach cancer concerning oesophageal cancer, there were more than 600,000 new cases of oesophageal cancer in 2020. After a successful resection in these cases, the role of early oral feeding (EOF) was questionable, due to the possibility of fatal anastomosis leakage. It is still debated whether EOF is more advantageous compared to late oral feeding. Our study aimed to compare the effect of early postoperative oral feeding and late oral feeding after upper gastrointestinal resections due to malignancy.

**Methods:**

Two authors performed an extensive search and selection of articles independently to identify randomized control trials (RCT) of the question of interest. Statistical analyses were performed including mean difference, odds ratio with 95% confidence intervals, statistical heterogeneity, and statistical publication bias, to identify potential significant differences. The Risk of Bias and the quality of evidence were estimated.

**Results:**

We identified 6 relevant RCTs, which included 703 patients. The appearance of the first gas (MD = −1.16; *p* = 0.009), first defecation (MD = −0.91; *p* < 0.001), and the length of hospitalization (MD = −1.92; *p* = 0.008) favored the EOF group. Numerous binary outcomes were defined, but significant difference was not verified in the case of anastomosis insufficiency (*p* = 0.98), pneumonia (*p* = 0.88), wound infection (*p* = 0.48), bleeding (*p* = 0.52), rehospitalization (*p* = 0.23), rehospitalization to the intensive care unit (ICU) (*p* = 0.46), gastrointestinal paresis (*p* = 0.66), ascites (*p* = 0.45).

**Conclusion:**

Early postoperative oral feeding, compared to late oral feeding has no risk of several possible postoperative morbidities after upper GI surgeries, but has several advantageous effects on a patient's recovery.

**Systematic Review Registration:**

identifier, CRD 42022302594.

## Introduction

Stomach cancer is the 5th most common cancer worldwide. It is the 4th most common cancer in men and the 7th most common cancer in women. There were more than 1 million new cases of stomach cancer in 2020. Concerning the stomach cancer, it causes 768,793/100,000 deaths worldwide. Esophageal cancer is the 8th most common cancer worldwide. It is the 7th most common cancer in men and the 13th most common cancer in women. There were more than 600,000 new cases of esophageal cancer in 2020. Esophageal cancer causes 544,076/100,000 deaths every year. Regarding tumors of the gastroesophageal junction, unfortunately we found little data. According to the latest 8th TNM classification, tumors of the gastroesophageal junction can be classified exactly as tumors of the stomach or stomach of the esophagus based on their location (1). After upper gastrointestinal surgeries, especially if the anastomosis is performed with the esophagus, the anastomosis failure rate is very high, reaching 9%–16% (2). For several decades, in upper GI resection surgeries in the postoperative period, inchoation of oral feeding was delayed to the seventh day in dread of occurring anastomosis insufficiency and generating systemic complications (3).

The human body produces up to 1 liter of saliva per day. This enzymatically active fluid, passes through the anastomosis, without triggering any anastomotic complication for the patient (4).

If the patient does not consume anything orally, the saliva is dense, its transit time increases, therefore it passes through the anastomosis slowly, possibly causing damage to the anastomosis.

Patients suffering from GI malignancies are often in an undernourished state. LOF (late oral feeding) protocol does not prove itself to be beneficial for the patient's nutritional state, while perioperative starvation provokes a severe catabolic state (5).

Enhanced recovery protocols for perioperative care, such as Enhanced Recovery After Surgery (ERAS), have gained wide acceptance. The concept of ERAS is to facilitate postoperative recovery and improve the quality of life. The postoperative oral feeding process is a fundamental component of the ERAS (6). EOF is defined by the start of oral feeding on the 1–3 postoperative days, while in the LOF feeding protocol, it starts 5–7 days after surgery. Despite several randomized clinical trials (RCTs) that have attempted to measure the benefits of EOF (early oral feeding), this protocol is not ubiquitously used. Early oral feeding (EOF) seems more profitable in the surgical profession to recover patients faster and decrease hospitalization time (7).

The aim is to compare the effect of early postoperative oral feeding and late oral feeding methods after upper gastrointestinal malignancy surgeries. For this express purpose, we performed a meta-analysis to compare the influence of the two diverse feeding strategies on postoperative recovery and to certify the safety and benefits of EOF.

We assume that early oral feeding does not increase the anastomotic insufficiency rate, nor the morbidity rate, while it has several beneficial effects on the general state and on the recovery time.

## Methods

A meta-analysis was carried out using the population-intervention-control-outcomes (PICO) format. Those studies were selected where patients had surgery because of upper GI malignancy (P), and postoperative feeding methods were compared (I and C). Mortality, complications, length of hospitalization, first flatus, and defecation were compared, as the outcomes of different treatment groups (O). The meta-analysis was reported in accordance with the Preferred Reporting Items for Systematic Review (PRISMA) statement and it was registered in advance in the PROSPERO database. The registration number is CRD 42022302594.

### Search strategy

The selection was conducted on electronic databases, including PubMed and Embase, and Cochrane. Restrictions were not applied. We started the search on the date of 1st of February 2021.

The search included the following keywords:

(((((upper GI OR upper gastrointestinal OR esophagus OR esophagus OR esophageal OR oesophageal OR stomach OR gastric) AND (surgery OR surgical OR operative OR operation OR resection)) OR (esophagectomy OR oesophagectomy OR gastrectomy)) AND ((enteral* OR oral*) AND (nutrition OR nutritional OR “oral feeding*” OR food))) AND random*.

### Inclusion and exclusion criteria

We searched for studies, involving patients with upper GI cancers, including oesophageal and gastric tumors, and we excluded all the cases when the surgery was performed because of benign diseases.

In our analysis, we compared the effect of early postoperative oral feeding, compared to late oral feeding, after upper gastrointestinal surgeries.

Articles were included if they provided data on at least two feeding modalities on patients with either EOF or LOF or both reporting the outcomes mentioned above. Only randomized controlled trials were included. Non-English language studies, studies focusing on pediatric cases, and studies with combined interventions were excluded.

### Selection process

The publications were processed by the EndNote X7.4 software (Clarivate Analytics, Philadelphia, PA, USA). Duplications were removed, and the remaining records were screened first by title, second by abstract, and finally by full-text by two independent authors (DLS and DB).

### Data extraction

Data were collected by two independent authors (DLS and AC) using an Excel (Office 365, Microsoft, Redmond, WA, USA) data sheet, based on predetermined criteria. Numerous binary variables outcomes were defined such as anastomosis insufficiency, pneumonia, wound infection, bleeding, ascites, rehospitalization, gastrointestinal paresis, and laryngeal nerve paresis. The appearance of the first gas, first postoperative defecation, and length of hospitalization were the outcomes of continuous variables.

### Statistical analysis

The statistical analyses were made with R (R Core Team) Software (8). For calculations and plots, we used the meta (9) and dmetar (10) packages.

For dichotomous outcomes the odds ratio (OR) with a 95% confidence interval (CI) was used for the effect measure; to calculate the OR, the total number of patients in each group and those with the event of interest were extracted from each study. Raw data from the selected studies were pooled using a random effect model with the Mantel-Haenszel method (11–13). For the pooled results exact Mantel-Haenszel method (no continuity correction) was used to handle zero cell counts (14). In individual studies, the zero cell count problem was adjusted by treatment arm continuity correction (15).

In the case of continuous outcomes, the mean differences (MD) with 95% CI were calculated as effect size. The extracted values to calculate the mean difference were the sample size (*N*), the mean, and the standard deviation (SD) in each group. If the mean and SD were not reported, the median and the upper and lower quartilee, the minimum and maximum values were extracted. If the mean value was not available, it was estimated from the sample size, median, and range using the method proposed by Luo et al. (16). Similarly, if the standard deviation was not reported, it was estimated from the sample size, median, and range using the method of Wan et al. (17). If the study number for the given outcome was over five, the Hartung-Knapp adjustment (18, 19) was applied (below six studies no adjustment was applied).

To estimate *τ*^2^ we used the Paule–Mandel method (20), and the *Q* profile method for calculating the confidence interval of *τ*^2^ (21).

Statistical heterogeneity across trials was assessed utilizing the Cochrane *Q* test, and the *I*^2^ values (22).

Forest plots and drapery plots (19, 23) were used to graphically summarise results. Where applicable we reported the prediction intervals (i.e., the expected range of effects of future studies) of results following the recommendations of IntHout et al. (19). A funnel plot of the logarithm of effect size and comparison with the standard error for each trial was used to evaluate publication bias. Publication bias was assessed with Egger's test using the Harbord method to calculate the test statistic (24).

Outlier and influence analyses were carried out following the recommendations of Harrer et al. (21) and Viechtbauer and Cheung (25).

### Quality assessment

To estimate the quality of the articles two independent authors (DLS and ACS) used the Risk of Bias Assessment Tool version 2 by Cochrane, and the GRADE approach was applied to assess the certainty of evidence.

## Results

**Figure 1:**
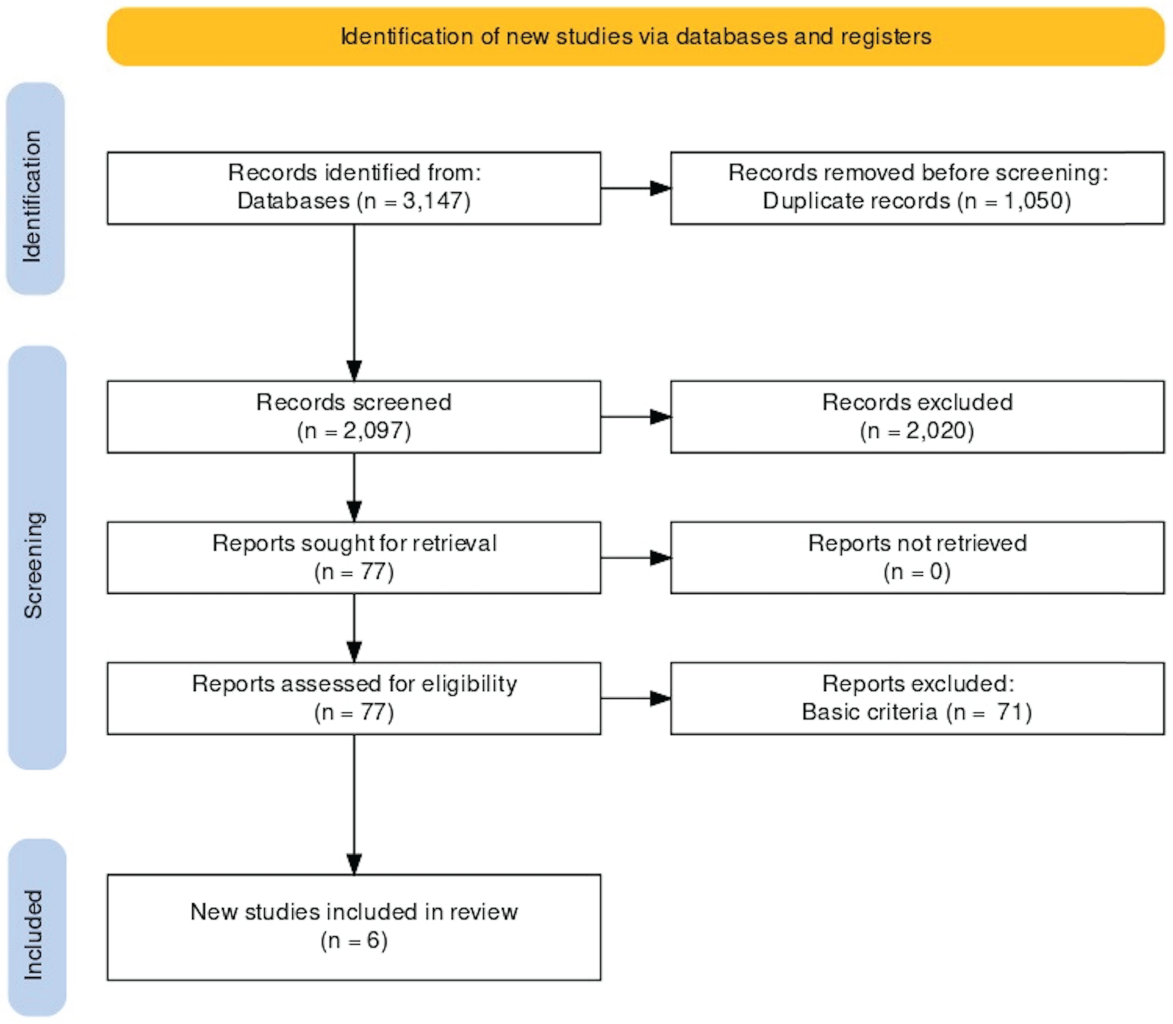
PRISMA flowchart.

We found 3,147 articles from Embase, Cochrane, and PubMed databases. We did not identify any additional articles from other sources. After the filter of duplication, title, and abstract, 77 articles remained. During the full-text filtering, we excluded 71 articles because they were not RCTs. We also excluded trials, which did not include patients with esophageal or gastric tumors and pediatric or animal experiments. We identified 6 relevant RCTs by full-text, which included 703 patients. The detailed steps of the selection process can be seen on the PRISMA flowchart (Figure 1).

### Characteristics of the studies

The details of the characteristics of the studies were shown in the table below (Table 1).

**Table 1:** Characteristics of the studies.

Author	Year of publication	Country	No of patients	Intervention	Surgery	Man/Woman	Age	Follow-up (mean)
Hur et al.	2011	Korea	54	GE	Open laparotomy	33/21	–	28 days
Mahmoodzadeh	2014	Iran	109	UGI	Transthoracic esophagectomy/total gastrectomy with Roux-en-Y/partial gastrectomy with Billroth I or II or Roux-en-Y	29/25	65, 3	–
Sun et al.	2018	China	86	EE	MIE McKeown	52/34	62, 4	–
Wang et al.	2019	China	100	GE	Total laparoscopic radical gastrectomy	71/29	54, 22	–
Shimizu et al.	2018	Japan	74	GE	Distal gastrectomy (DG)	137/79	65, 45	–
					Total gastrectomy (TG)			
Sun et al.	2017	China	280	EE	MIE McKeown	195/85	63	24 weeks

### Bowel movement

**Figure 2:**
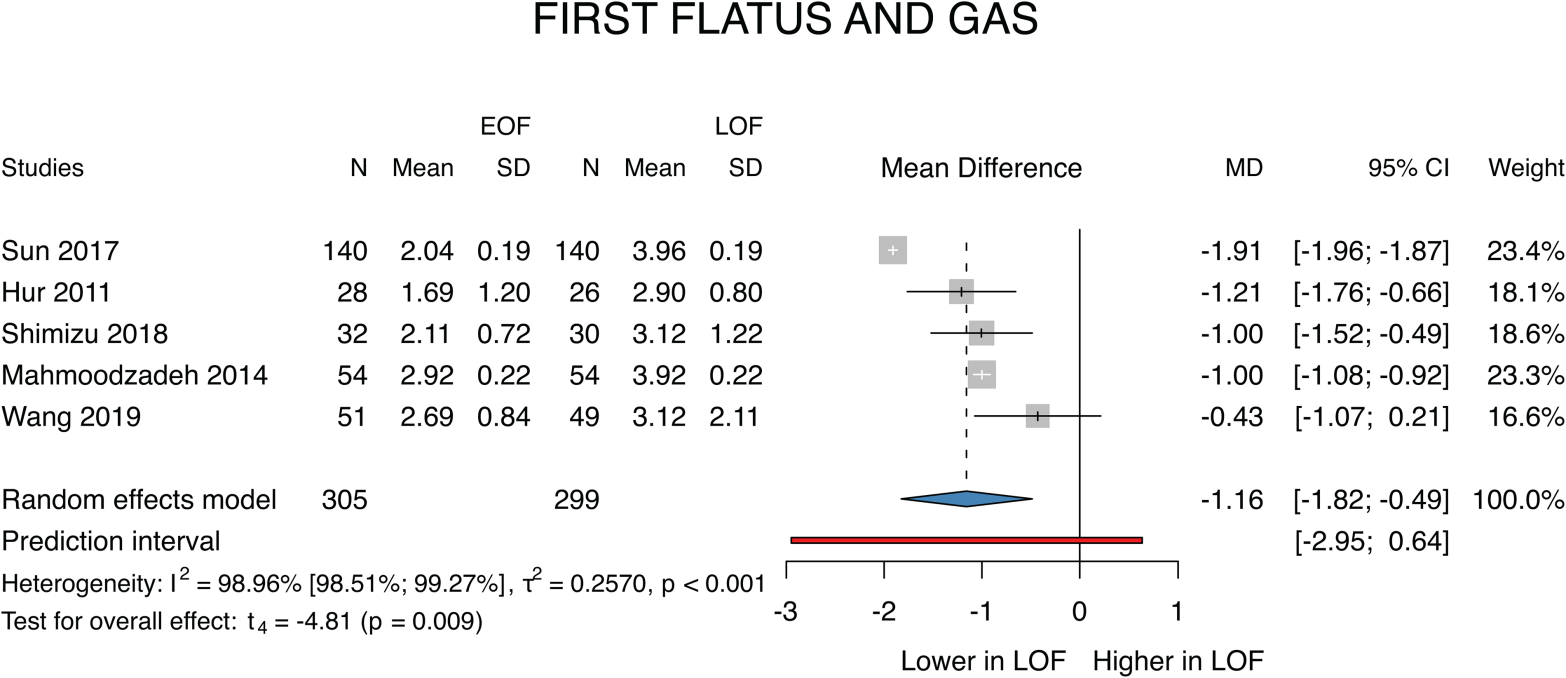
First flatus and gas.

In the case of the first flatus or gas, a total of 5 studies (26–30) were selected for analyses covering a total of 604 patients. We found that the first flatus and gas appeared earlier in the EOF group (MD: −1.16; *p* = 0.009; 95% CI: [−1.82; −0.49]). The between-study heterogeneity was significant (*I*^2 ^=^ ^99%; *p* < 0.001) (Figure 2).

**Figure 3:**
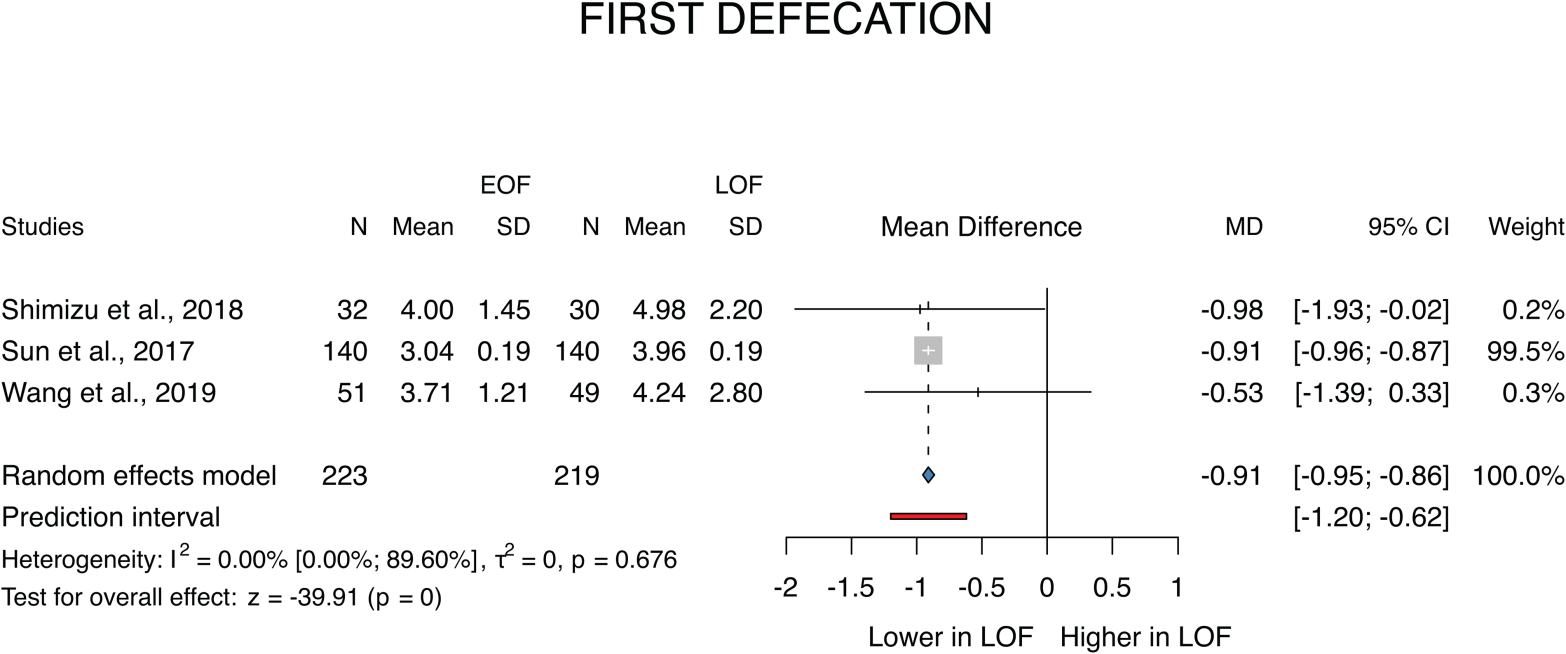
First defecation.

A total of 3 studies (26, 28, 30) were selected for the analyses of the first defecation covering a total of 442 patients. We found that first defecation appeared significantly earlier in the EOF group (MD: −0.91; *p* < 0.001; 95% CI: [−0.95; −0.86]). The between-study heterogeneity was not significant (*I*^2 ^= 0%; *p* = 0.676) (Figure 3).

### Length of hospital stay

**Figure 4:**
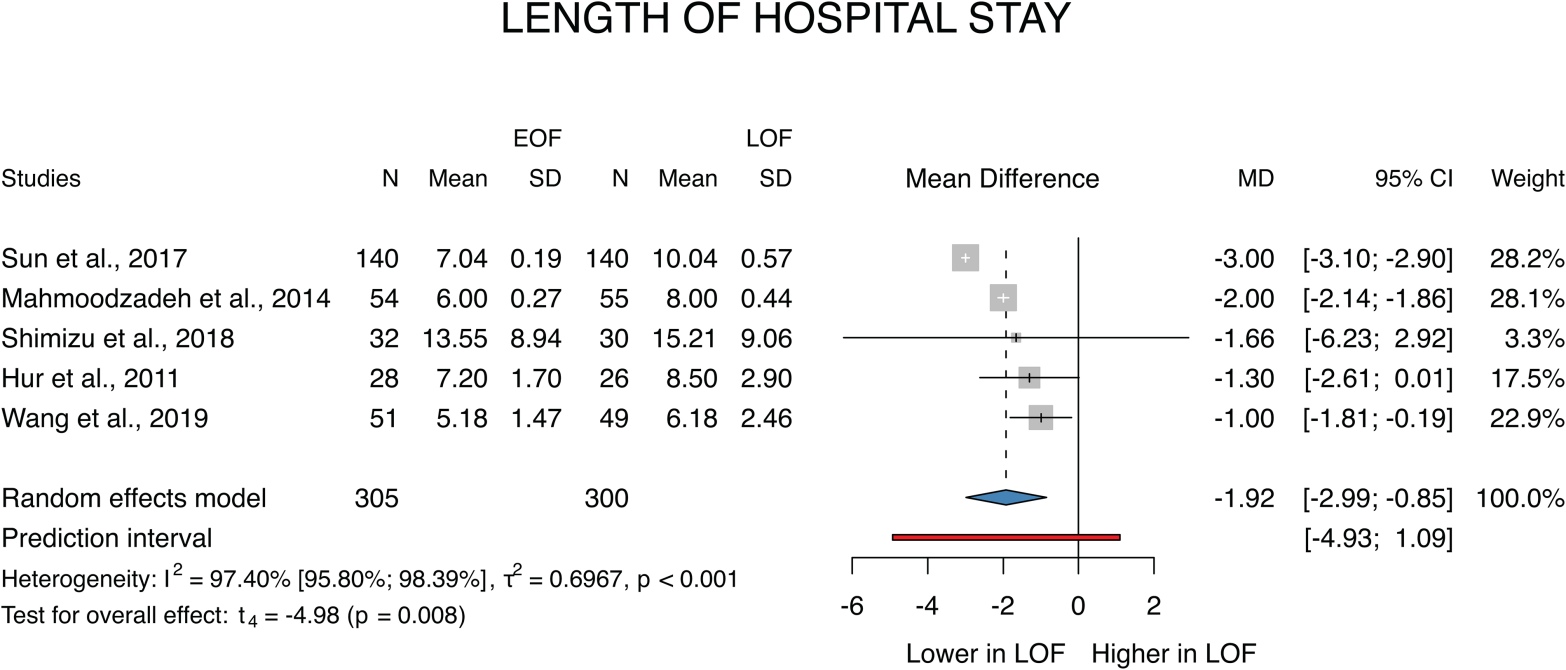
Length of hospital stay.

A total of 5 (26–30) studies were selected for analyses covering a total of 605 patients. We found that the first flatus and gas appeared earlier in the EOF group (MD: −1.92; *p* = 0.008; 95% CI: [−2.99; −0.85]). The between-study heterogeneity was significant (*I*^2 ^= 97%; *p* < 0.001) (Figure 4).

### Rehospitalization

A total of 5 studies (26–29, 31) were selected for analyses covering a total of 603 patients. We found that there is no statistically significant difference between the two groups (OR = 0.57; *p* = 0.25; 95% CI: [0.18; 1.80]). The between-study heterogeneity was not significant (*I*^2 ^= 0%; *p* = 0.47).

### Adverse events

#### Anastomosis leakage

**Figure 5:**
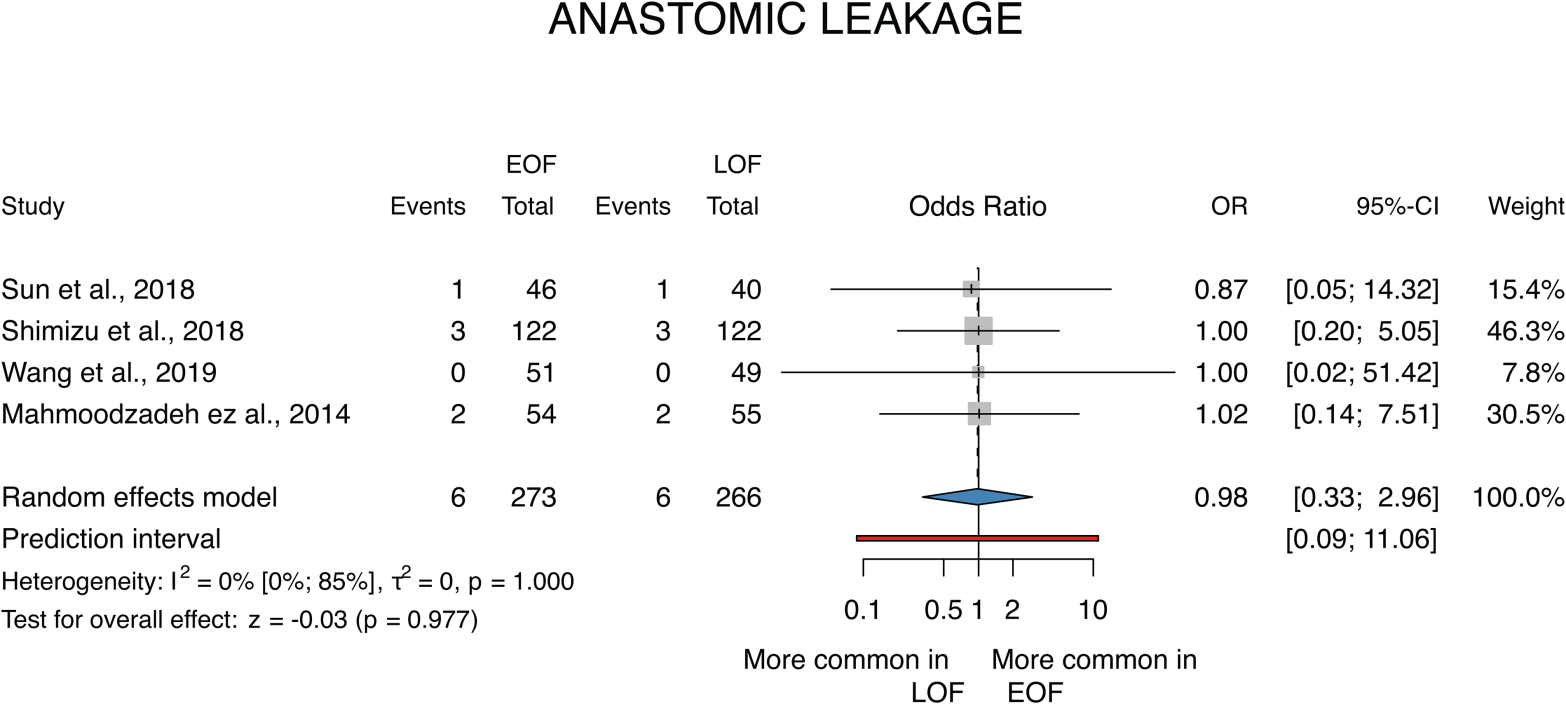
Anastomotic leakage.

A total of 4 studies (28–31) were selected for analyses covering a total of 539 patients. We found that there is no statistically significant difference between the two groups (OR = 0.98; *p* = 0.98; 95% CI: [0.33; 2.96]). The between-study heterogeneity was not significant (*I*^2 ^= 0%; *p* = 0.01) (Figure 5).

#### Pneumonia

A total of 4 studies (26, 28, 29, 31) were selected for analyses covering a total of 549 patients. We found that there is no statistically significant difference between the two groups (OR = 0.95; *p* = 0.88; 95% CI: [0.51; 1.79]). The between-study heterogeneity was not significant (*I*^2 ^= 0%; *p* = 0.92).

#### Wound infection

A total of 4 studies (26, 27, 30, 31) were selected for analyses covering a total of 520 patients. We found that there is no statistically significant difference between the two groups (OR = 1.59; *p* = 0.48; 95% CI: [0.44; 5.77]). The between-study heterogeneity was not significant (*I*^2 ^= 0%; *p* = 0.85).

#### Bleeding

A total of 4 studies (26–28, 30) were selected for analyses covering a total of 508 patients. We found that there is no statistically significant difference between the two groups (OR = 1.70; *p* = 0.52; 95% CI: [0.34; 8.61]). The between-study heterogeneity was not significant (*I*^2 ^= 0%; *p* = 0.92).

#### Ascites

A total of 3 studies (26, 27, 31) were selected for analyses covering a total of 420 patients. We found that there is no statistically significant difference between the two groups (OR = 0.56; *p* = 0.449; 95% CI: [0.12; 2.52]). The between-study heterogeneity was not significant (*I*^2 ^= 0%; *p* = 0.82).

#### Gastrointestinal paresis

A total of 3 studies (27, 28, 30) were selected for analyses covering a total of 228 patients. We found that there is no statistically significant difference between the two groups (OR = 0.55; *p* = 0.43; 95% CI: [0.12; 2.47]). The between-study heterogeneity was not significant (*I*^2 ^= 0%; *p* = 0.53).

#### Recurrent laryngeal nerve injury

A total of 3 studies (26, 29, 31) were selected for analyses covering a total of 475 patients. We found that there is no statistically significant difference between the two groups (OR = 0.96; *p* = 0.9; 95% CI: [0.51; 1.82]). The between-study heterogeneity was not significant (*I*^2 ^= 0%; *p* = 0.99).

### Risk of bias

ROB was assessed as low in all outcomes (26–31). The detailed estimation results are summarised in the table below (Table 2).

**Table 2:** D1, randomisation process; D2, deviations from the intended interventions; D3, missing outcome data; D4, measurement of the outcome; D5, selection of the reported result.

Outcome	ID	D1	D2	D3	D4	D5	Overall
First flatus and gas	Sun et al., 2017	Low	Low	Low	Low	Low	Low
	Hur et al., 2011	Low	Low	Low	Low	Low	Low
	Shimizu et al., 2018	Low	Low	Low	Low	Low	Low
	Mahmoodzadeh et al., 2014	Low	Low	Low	Low	Low	Low
	Wang et al., 2019	Low	Low	Low	Low	Low	Low
First defecation	Shimizu et al., 2018	Low	Low	Low	Low	Low	Low
	Sun et al., 2018	Low	Low	Low	Low	Low	Low
	Wang et al., 2019	Low	Low	Low	Low	Low	Low
Length of hospital stay	Sun et al., 2017	Low	Low	Low	Low	Low	Low
	Mahmoodzadeh et al., 2014	Low	Low	Low	Low	Low	Low
	Shimizu et al., 2018	Low	Low	Low	Low	Low	Low
	Hur et al., 2011	Low	Low	Low	Low	Low	Low
	Wang et al., 2019	Low	Low	Low	Low	Low	Low
Rehospitalization	Mahmoodzadeh et al., 2014	Low	Low	Low	Low	Low	Low
	Hur et al., 2011	Low	Low	Low	Low	Low	Low
	Sun et al., 2017	Low	Low	Low	Low	Low	Low
	Sun et al., 2018	Low	Low	Low	Low	Low	Low
	Shimizu et al., 2018	Low	Low	Low	Low	Low	Low
Anastomotic leakage	Sun et al., 2018	Low	Low	Low	Low	Low	Low
	Shimizu et al., 2018	Low	Low	Low	Low	Low	Low
	Wang et al., 2019	Low	Low	Low	Low	Low	Low
	Mahmoodzadeh et al., 2014	Low	Low	Low	Low	Low	Low
Pneumonia	Sun et al., 2017	Low	Low	Low	Low	Low	Low
	Mahmoodzadeh et al., 2014	Low	Low	Low	Low	Low	Low
	Sun et al., 2018	Low	Low	Low	Low	Low	Low
	Shimizu et al., 2018	Low	Low	Low	Low	Low	Low
Wound infection	Sun et al., 2018	Low	Low	Low	Low	Low	Low
	Wang et al., 2019	Low	Low	Low	Low	Low	Low
	Sun et al., 2017	Low	Low	Low	Low	Low	Low
	Hur et al., 2011	Low	Low	Low	Low	Low	Low
Bleeding	Hur et al., 2011	Low	Low	Low	Low	Low	Low
	Shimizu et al., 2018	Low	Low	Low	Low	Low	Low
	Wang et al., 2019	Low	Low	Low	Low	Low	Low
	Sun et al., 2017	Low	Low	Low	Low	Low	Low
Ascites	Hur et al., 2011	Low	Low	Low	Low	Low	Low
	Sun et al., 2017	Low	Low	Low	Low	Low	Low
	Sun et al., 2018	Low	Low	Low	Low	Low	Low
Gastrointestinal paresis	Hur et al., 2011	Low	Low	Low	Low	Low	Low
	Wang et al., 2019	Low	Low	Low	Low	Low	Low
	Shimizu et al., 2018	Low	Low	Low	Low	Low	Low
Recurrent laryngeal nerve injury	Sun et al., 2017	Low	Low	Low	Low	Low	Low
	Mahmoodzadeh et al., 2014	Low	Low	Low	Low	Low	Low
	Sun et al., 2018	Low	Low	Low	Low	Low	Low

### Grade

The quality of the evidence was estimated as moderate in all outcomes (26–31) because most articles originated from Asia, therefore we cannot standardize the results. The results of the GRADE were contained in the table below (Supplementary Table S1).

## Discussion

In the case of operations performed for upper gastrointestinal tumors, the mortality and morbidity rates are very high, especially if the anastomosis is performed with the esophagus (2).

For decades, anastomosis failure was one of the most dangerous complications leading to other morbidities. Li et al. (32) described that EOF can increase the anastomosis leakage rate during open surgery, however, they worked with a small number of cases. Fearing this complication, the “nil per os” feeding method spread (3), however nowadays, MIE operations have become more common, because of several advantages (33), including the chance of anastomotic leakage does not increase during the EOF (32).

In recent years, it has been proven that the ERAS protocol has a beneficial effect on the prehabilitation and rehabilitation of patients, which includes early oral feeding after surgery as part of the multimodal care protocol (6), therefore the topic of EOF is becoming increasingly popular in literature. Previously 4 meta-analyses (32, 34–36) dealt with the comparative study of early and late oral feeding. They found the EOF is feasible and safe, especially in the case of MIE, however, they have some limitations, such as the small number of included studies, high heterogeneity between the groups, and complications that were not discussed in detail, therefore we investigate the topic again.

We prepared a meta-analysis based on the PRISMA protocol, in which we included 6 studies with the participation of 703 patients. In these studies, early (EOF) and late (LOF), oral feeding methods were used after oesophageal and gastric cancer surgeries, and then the results of the 2 groups were compared. In the EOF group, they were allowed to consume liquid on the second post-operative day orally, and then from the postoperative day on, they started giving formula, which is how the feeding method is structured. In the control group, for 5–7 days after the operation, the patients were not allowed to consume food orally, it was provided enterally or by other parenteral means.

As previously described, anastomotic leakage is one of the most common complications, associated with life-threatening infection and mortality, and influences the response of therapy, therefore, it is one of the most important outcomes. There was no significant difference between the EOF and LOF groups, based on our study. Li et al. (32) also found no significant difference in their meta-analysis between the two major groups. Because of the high heterogeneity, they performed subgroup analysis. This result, due to the small number of elements, should be addressed with some concerns. In the MIE subgroup, they found no difference, however, in the case of open surgery, the EOF can be associated with a higher risk of anastomosis leakage. The effect of the EOF depends on the site of the anastomosis. In the case of cervical anastomosis, the EOF can be at higher risk, however, in the thoracic subgroup, there was no significant difference (32). In gastric cancer surgery, Liu et al. found no difference between the EOF and the LOF group (35).

In the case of gastric cancer, He (34) and Liu et al. (35), found no difference in the case of overall complications, however, Xin et al. found that the EOF decreased the risk of postoperative complications (36). We investigated the postoperative complications of upper GI surgery separately, and we found no difference in bleeding (*p* = 0.52), wound infection (*p* = 0.48), ascites (*p* = 0.45), and gastrointestinal paresis (*p* = 0.43). He et al. also found no significance in feeding intolerance (0.62) (34).

However, we do not investigate the question due to a lack of data, He (34) and Xin et al. (36) found EOF can increase nutrition values, albumin (*p* < 0.0001), and prealbumin (*p* < 0.001) levels in case of gastrectomies. Xin et al. found a significant increment of immune indicators like CD3+ (*p* = 0.0009), CD4+ (*p* < 0.00001), CD4+/CD8+ (<0.00001), and NK cells (<0.00001) under the influence of EOF (36).

The appearance of the first flatus and gas is earlier in EOF, based on our investigation (*p* = 0.009; MD = −1.16 [−1.82; −0.49]), which is confirmed by He (34) and Liu et al. (<0.0001) (35), and we also found the first defecation comes earlier in EOF (*p* < 0.001, MD = −0.91 [−0.95; −0.86]).

The main advantage of applying the EOF is the shorter length of hospital stay, which our investigation (*p* = 0.008, MD = −1.92 [−2.99; −0.85]), and the meta-analyses by He (34) and Liu et al. (35) also confirmed (*p* < 0.001). Even though patients can be discharged earlier, the rate of rehospitalization does not increase (*p* = 0.25).

We found a lack of data, but logically the cost of hospitalization can decrease significantly, which He et al. also verified (MD: −4.21, *p* < 0.001) (34). Altman et al. examined the elements of the ERAS protocol and concluded that it can reduce hospital stay time and costs (1). Liu et al. also found that EOF can decrease the hospitalization cost (*p* = 0.014) (35) and Wang et al. estimated the difference at about 2,000 yuan (300 USD), however, the significance was not verified (30). An important element in reducing hospital stay is the length of stay in the intensive care unit, which can be reduced to a significant extent by starting oral feeding early, compared to the late-started feeding group (37).

Lower hospital costs can be achieved by reducing the length of stay in the intensive care unit. Roh et al. analyzed the length of hospital stay after a minimally invasive subtotal gastrectomy. In this study, the hospital length of stay in the early feeding group was significantly lower than that in the LOF group. However, the complication rate was not found to be higher in the EOF group (38).

In our analysis, we did not examine mortality as an outcome due to the small amount of available data, despite the fact that we planned to examine it in advance. A short-term 30-day follow-up of mortality was performed by Jang et al. (7) who found no difference in the mortality rate, however, no long-term follow-up was done in terms of this outcome. A 30-day follow-up was also carried out in the study published by Hur et al. (27), mortality as an outcome shows a long-term improving trend in the early feeding group, because the improvement of mortality indicators, such as acute phase proteins and the decreasing sepsis rate, reduce morbidity and thus mortality indicators. It can be said that the mortality rate can be indirectly reduced by using early oral feeding, which can be achieved through the reduction of morbidity factors and cannot be interpreted directly as an effect of oral feeding.

Quality of life is a very important aspect in addition to postoperative morbidities, although we could not analyze it due to the small sample size and the high heterogeneity of the data, therefore it would be useful to measure QOL with one standard method, for example using the EORTC QLQC30 score system, in future studies.

Patients who have undergone upper GI surgery are often malnourished, which is also contributed to by the surgical metabolic stress. Weight loss and the weakening of the patient's physical condition have been shown an increased the mortality rate (39). Pre- and post-operative weight loss and body mass index have an impact on prognosis in patients with oesophageal cancer (39).

At the same time, this is also a factor, affecting the quality of life, which can be significantly improved by starting early oral feeding. Yang et al. investigated the effect of early oral feeding on the quality of life of patients who underwent minimally invasive oesophagectomy. They used Cancer-Quality of life Question-Core (QLQ-C30, version 3) and Oesophageal Cancer Module (QLQ-OES-18) questionnaires. They found that weight loss can be reduced and has a positive effect on early recovery, and can demonstrably improve the quality of life (40).

In the future, it would be necessary to widely use quality-of-life questionnaires as part of the ERAS protocol for patients undergoing upper gastrointestinal surgery. For example, Sun et al. used the EORTC QLQC30 questionnaire to assess the quality of life, it can be said that the EOF group had significantly better results compared to the LOF group (26).

### Strength

We selected high-quality articles as there were only randomized controlled trials selected, therefore the risk of bias is low.

The definition of outcomes is homogenous, thereby increasing the quality.

The characteristic of patients was similar in the EOF and the LOF group.

### Limitation

We were primarily interested in examining the EOF during oesophageal surgery, but unfortunately, due to the small number of RCTs, we had to combine it with gastrectomy, so in the end, we examined an integrated UGI group. Due to the rigorous criteria, a small number of cases were available. Another limitation is the averages had to be estimated in many places because it was not described precisely in the articles, and median values were not given in many places.

Mostly Asian and American articles were included, therefore the population of the patients was overwhelmingly Asian, while European and American were represented by only one article each. Thus, these results are only applicable to the European and American populations in a limited manner.

Due to the small number of cases and few studies, we did not separate the results of gastric tumor and oesophageal tumor patients during our meta-analysis but examined them in one group.

### Implication for research

Recently, more and more articles deal with the advantages of EOF, but the number of RCTs is still small. In our meta-analyses some limitations emerge, therefore further large sample size randomized controlled investigation is needed in the topic of the esophagus and gastric resection, especially in cases of minimally invasive UGI surgery. Trials should originate from distinct countries so that the results can be standardized. This is the reason why we are planning on conducting a multicentric clinical research project involving multiple Hungarian medical institutions that handle UGI surgeries.

### Implication for practice

In our meta-analysis, we proved that the use of EOF has many advantages, but does not involve significant complications. It reduces the length of hospital stay and contributes to a better immune status, which in itself reduces the development of postoperative complications and contributes to a faster recovery time. Anastomotic leakage can be a dangerous complication in connection with EOF, but we could not prove this risk. All in all, we can say that EOF has negligible risk, however, it is a safe way to improve the recovery of patients.

## Conclusion

Our meta-analysis is more comprehensive and accurate than before, due to rigorous criteria. In conclusion, it can be said that oral feeding started early after surgery is safe even after upper gastrointestinal surgery. Based on our results EOF does not associate with higher morbidity especially anastomotic leakage, pneumonia, wound infection, bleeding, ascites, gastrointestinal paresis, and recurrent laryngeal nerve injury. The main advantages of the EOF are the appearance of the first flatus and defecation earlier, which means the recovery time of bowel function is more rapid. The risk of rehospitalization was similar in the investigated groups, and the time of hospital stay is also shortened in the EOF, which magnetifies lower cost. Even though many studies are still needed on this topic in the future, based on our results, we recommend the usage of EOF after upper GI surgery in practice, especially within the framework of the ERAS protocol, due to its many advantages and negligible complications.

## Data Availability

The raw data supporting the conclusions of this article will be made available by the authors, without undue reservation.

## Supplementary material

Supplementary Figure 1

Rehospitalization.

Supplementary Figure 2

Pneumonia.

Supplementary Figure 3

Wound infection.

Supplementary Figure 4

Bleeding.

Supplementary Figure 5

Ascites.

Supplementary Figure 6

Gastrointestinal paresis.

Supplementary Figure 7

Recurrent laryngeal nerve (RLN) injury.
